# Influence of Glassy Carbon Surface Finishing on Its Wear Behavior during Precision Glass Moulding of Fused Silica

**DOI:** 10.3390/ma12050692

**Published:** 2019-02-26

**Authors:** Tim Grunwald, Dennis Patrick Wilhelm, Olaf Dambon, Thomas Bergs

**Affiliations:** 1 Chair for Manufacturing Technology, Department of Forming, Tool Machine Laboratory (WZL) of RWTH Aachen University, 52074 Aachen, Germany; t.bergs@wzl.rwth-aachen.de; 2 Department of Fine Machining and Optics, Fraunhofer Institute for Production Technology IPT, 52074 Aachen, Germany; dennis.patrick.wilhelm@ipt.fraunhofer.de (D.P.W.); olaf.dambon@ipt.fraunhofer.de (O.D.)

**Keywords:** Glassy Carbon, surface finishing, polishing, Precision Glass Moulding, Fused Silica, White Light Interferometry, atomic force microscopy, wear, adhesion

## Abstract

Laser technology has a rising demand for high precision Fused Silica components. Precision Glass Moulding (PGM) is a technology that can fulfil the given demands in efficiency and scalability. Due to the elevated process temperatures of almost 1400 °C and the high mechanical load, Glassy Carbon was qualified as an appropriate forming tool material for the moulding of Fused Silica. Former studies revealed that the tools’ surface finishing has an important influence on wear behaviour. This paper deals with investigation and analysis of surface preparation processes of Glassy Carbon moulds. In order to fulfil standards for high precision optics, the finishing results will be characterised by sophisticated surface description parameters used in the optics industry. Later on, the mould performance, in terms of wear resistance, is tested in extended moulding experiments. Correlations between the surface finish of the Glassy Carbon tools and their service lifetime are traced back to fundamental physical circumstances and conclusions for an optimal surface treatment are drawn.

## 1. Introduction

Production systems with laser beam sources are becoming increasingly powerful and tend towards more compact designs [1]. But only a few materials for optics for beam shaping can withstand the high thermal loads permanently [2]. For this reason, we are researching manufacturing processes with which lenses made of resistant Fused Silica can be produced more cost-effectively and according to high quality standards.

**Figure 1:**
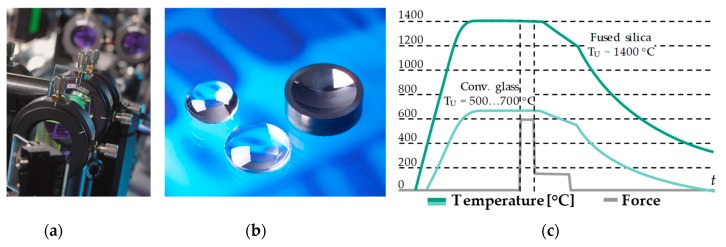
(**a**) Beam shaping optics in laser systems; (**b**) Aspherical Fused Silica lenses produced by PGM; (**c**) PGM process chart of Fused Silica moulding (PGM, Precision Glass Moulding).

The Institute is investigating the manufacturing of these high-precision Fused Silica optics using the Precision Glass Moulding process (PGM) (Figure 1) [3]. Optics, which were traditionally produced by multi-step grinding and polishing processes, are thus produced in just one process step. Due to the replicative character of PGM, even complex geometries can be realised efficiently [4]. In this publication, the investigation of contact behaviour between tool and glass in Fused Silica moulding is the main focus.

Optics made of Fused Silica enjoy a high industrial demand. Due to its outstanding properties, such as the high transmission range from 185 nm to 2.5 μm regarding electromagnetic radiation, a high homogeneity and a very good temperature resistance, it offers excellent conditions for special applications [2,5]. Conventionally, optics made of this glass type are ground and polished, in some cases pursued with even more sophisticated machining technology such as Magneto-rheological Finishing (MRF) or Ion Beam Figuring (IBF) [6,7,8]. In order to form Fused Silica glass during precision moulding, it is heated up to 1360 °C. For this reason, our department is researching the use of the high-tech material Glassy Carbon as a corresponding forming tool material. Glassy Carbon offers exceptionally high thermal and mechanical load resistance in vacuum or inert gas [9,10,11].

Despite extreme resistance to temperature and mechanical stress, wear and tear can be seen on the surface after several cycles of the Fused Silica PGM process. These signs of wear exist due to various wear mechanisms. The growth of the defects is facilitated by repeated pressing processes. Not only tool are material chippings a problem but also Fused Silica, which adheres to the already existing chippings and micro hills, which creates adhesion between the two materials. The common way to reduce wear in PGM, that is, the application of a protective coating, cannot be applied to Fused Silica Moulding because of the extreme temperature conditions [12,13,14].

Therefore, the aim of the research is to understand the causes of defect formation during Fused Silica forming (and thus to guarantee the specified quality of the Fused Silica optics). In particular, this paper focuses on the investigation of the influence of the Glassy Carbon tool’s surface finish on its wear behaviour in Fused Silica PGM.

## 2. Method, Materials and Processes

### 2.1. Method

**Figure 2:**
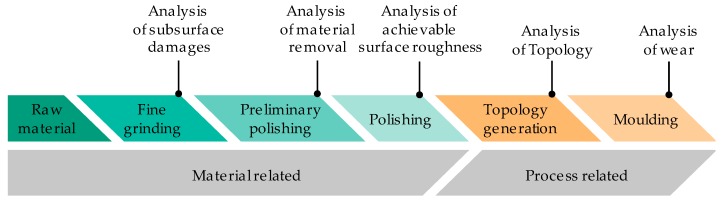
Approach used for studying the wear behaviour of Glassy Carbon forming tools in Fused Silica PGM.

In order to study the influence of the Glassy Carbon tools’ surface finish on its wear behaviour during Fused Silica moulding, a multi-step approach it used (Figure 2). At first, material related issues are discussed. A detailed analysis of the microstructure and subsurface damage during grinding of Glassy carbon is carried out and followed by polishing experiments. The relationship between material removal and the choice of polishing abrasives is discussed.

Subsequently, the material-related findings are used to answer process-related issues. The preliminary polishing experiments give hints of the manufacturing of different surface topologies. These surface topologies are needed to study the influence of the tools’ surface finishings on their wear behaviour during glass replication.

The following paragraphs in this section give further information on the materials, machines and processes used to gain the results that are presented later on.

### 2.2. Fused Silica as Optical Material

**Figure 3:**
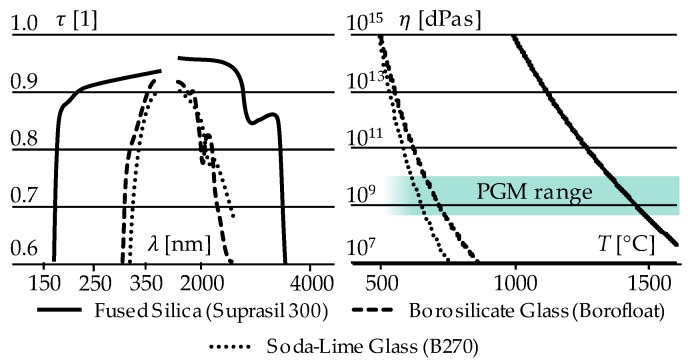
(**Left**) Comparison of different glass types in terms of transmissivity *τ* over wavelength *λ*; (**Right**) Comparison of different glass types in terms of viscosity *η* over temperature *T* [5,16,17].

In recent years, laser sources became increasingly powerful. This implies high loads on optical materials that are used for beam shaping and guidance. Conventional glass types, such as soda-lime or borosilicate glass, cannot be used for laser powers above ca. 1 kW. Their internal absorbance (followed by warming and alternation of the refractive index) as well as the following thermal expansion lead to a “focus shift” that impairs the performance of the optical system [2,15]. A complete failure due to glass breakage (the releasing of mechanical stresses induced by thermal expansion) can destroy the entire system. Fused Silica as the purest kind of amorphous SiO_2_ neither has any foreign atom inclusions nor cut-off points within its network leading to high broadband transmission as well as high thermal stability (Figure 3, Table 1).

For this study, the type Suprasil 300, provided by Heraeus, was selected because of its extremely low OH^−^-impurity content. This qualifies that this glass type for high power IR-applications and parasitic chemical interactions during moulding induced by outgassing can be neglected.

### 2.3. Glassy Carbon as a Tool Material for Precision Glass Moulding

Glassy Carbon is mainly based on sp^2^ bonds but it also contains in-plane defects and features variable bond-lengths. The sp^2^ orbitals form a symmetrical hexagonal arrangement aligned on one plane. The close arrangement of the Glassy Carbon layer network consists of flat carbon hexagons. More detailed investigations of Glassy Carbon with transmission electron microscopes (TEM) show that the microstructure consists of crumpled sp^2^-hybridised carbon layers, including micropores of about 1–5 nm in diameter [18].

**Figure 4:**
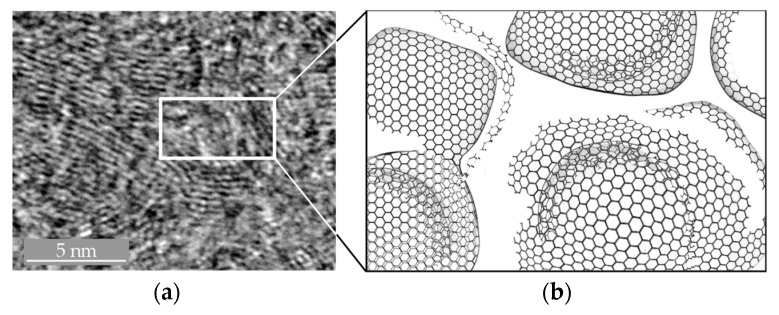
(**a**) Microstructure derived from transmission electron microscope (TEM)-measurement performed at Fraunhofer Institute for Microstructure of Materials and Systems (IMWS) with commercially available Glassy Carbon; (**b**) Model-based visualisation of microstructure according to Harris [18].

Figure 4a is a TEM image obtained during our experiments, and Figure 4b shows a simulated microstructural model of glassy carbon suggested by Harris [18].

If certain polymers are carbonised under carefully controlled conditions, a carbon with a glass-like structure is formed. Carbonisation is based on the principle of pyrolysis. Thermal decomposition of chemical compounds takes place under exclusion of oxygen. All elements, with the exception of carbon, are removed from the structure. What remains is a brittle and hard material, which cannot be converted into graphite by the application of high temperatures [19]. Due to its glassy appearance and fracture behaviour, this carbon is similar to glass in its physical properties and is therefore called Glassy Carbon.

This type of carbon offers a lot of particular properties. Glassy Carbon offers excellent resistance to gases in direct comparison to graphite. This value is comparable to that of glassbut it is not only this property that distinguishes Glassy Carbon as a material. Good electrical conductivity (42 × 10^−6^ Ωm), high resistance to thermal shock and good corrosion resistance also highlight it. The comparably high stiffness and increasing tensile strength under temperature load both contribute to good thermo-mechanical resistance [20]. When heated, the material expands only slightly ($\alpha_{GC}$ ~ 2.0–2.2 × 10^−6^ 1/K). Under inert gas the high temperature resistance can be up to 3000 °C. Furthermore, this material behaves isotropically in comparison to other carbon forms. Low reactivity with chemical substances makes it very robust. The most important properties of Glassy Carbon as a forming tool for precision moulding of Fused Silica glass are its high temperature and corrosion resistance [9,10,11,21,22].

In order to qualify Glassy Carbon as a tool material for PGM, the generation of an optical surface quality (“mirror-like”) is mandatory. Recent machining of Glassy Carbon is carried out by grinding, polishing and etching [19,23]. The material used in this study was made available by Tokai Carbon K.K. (Minato-ku (Tokio), Japan), quality grade GC30 [9].

### 2.4. Machining Setup and Process

**Figure 5:**
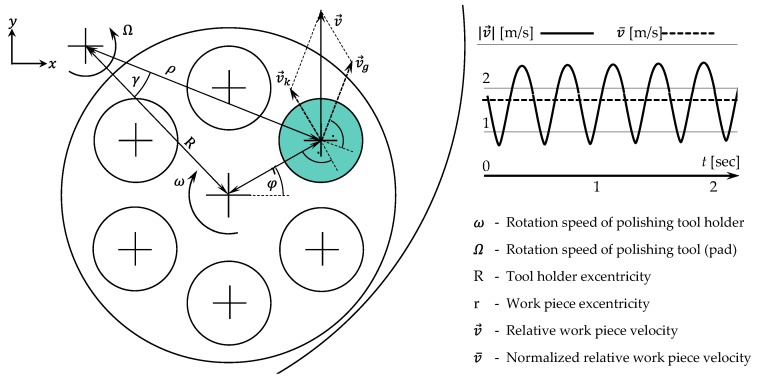
Kinematical analysis of polishing experiments using a flat polishing machine.

Both grinding and polishing were carried out on a Bühler Phoenix 4000 flat polishing machine (Bühler AG, Uzwil, Switzerland). The setup includes a rotation of the polishing pad (*Ω*) and an independent rotation of the workpiece holder (*ω*) (Figure 5). This kinematic enables different polishing paths, such as cycloid or hypocycloid.

For the preliminary evaluation of polishing processes, the Preston hypothesis is usually applied (1) [24]. It expresses the time-dependant material removal *dz/dt* as the product of contact pressure *p* between workpiece and polishing pad, the relative velocity $\vec{v}$ between the same contact partners as well as the empirical constant *K* that comprises all other process influences such as polishing, abrasive, chemical interactions and so forth. By keeping all process parameters and surrounding circumstances constant, a direct comparison of the abrasives can be derived by means of *K* (see Section 3.2).

(1) $$\frac{dz}{dt}=K\cdot p\cdot \vec{v}$$

Since the contact pressure is a directly manipulatable variable, the relative velocity $\vec{v}$ is determined by the rotations *Ω* as well as *ω* and can be calculated by the following expression (2). For better handling, the cumulated velocity $\bar{v}$ is used subsequently (3).

(2) $$\vec{v}=\left(\begin{array}{c} {\vec{v}}_{x}\\ {\vec{v}}_{y} \end{array}\right)={\vec{v}}_{g}+{\vec{v}}_{k}\;\vec{v}=\omega r\left(\begin{array}{c} -\sin\varphi \\ \cos\varphi \end{array}\right)+\Omega R\left(\begin{array}{c} -\cos\gamma \\ -\sin\gamma \end{array}\right)|\gamma =\arcsin\left(\frac{r}{\rho }\sin\left(\frac{\pi }{2}-\varphi \right)\right),\;\rho =R^{2}+r^{2}-2Rr\cdot \cos\left(\frac{\pi }{2}-\varphi \right)$$

(3) $$\bar{v}=\int \left|\vec{v}\right|dt$$

The grinding and polishing specimens made of Glassy Carbon are disc-shape with a diameter of 34 mm. After cleaning and surface qualification, the same samples can be installed directly into a commercially available glass moulding machine.

### 2.5. Precision Glass Moulding of Fused Silica

The next coming description explains the glass moulding process gradually (also see Figure 1a).

- After surface finishing and cleaning, the moulding tools are integrated into moulding dies and fixed in the machine (upper and lower mould); a dedicated glass preform is placed on the lower mould.
- The moulding process starts with an evacuation of the moulding chamber for preventing an oxidation of the moulding tools. The evacuation is followed by a nitrogen purge.
- After this evacuation, the glass preform and the moulding tools are heated up to 1360 °C by using infrared lamps.
- Subsequent to reaching a temperature of about 1360 °C a four-minute soaking phase starts, the soaking effectuates a homogeneous temperature propagation.
- In the following, a four-minute moulding phase is starting. Due to a 2 kN moulding force that the Fused Silica is pressed into the desired shape.
- When the lens is shaped, a two-step cooling phase begins. The first step cools the temperature gradually down to 600 °C by using nitrogen flow. The nitrogen effects a thermal convection. At 600 °C the flow increases. A faster cooling rate is the result.
- At a temperature of 200 °C the process is completed. The moulding tools drift apart and the moulded glass component can be unloaded by means of vacuum grippers.

The experiments are carried out on a Toshiba GMP 207HV (Toshiba Machine Co. Ltd., Numazu (Shizuoka), Japan). The moulding cycles are repeated with constant set of parameters until a development of wear is observable. In this case, 30 moulding cycles were carried out.

## 3. Results

### 3.1. Fine Grinding of Glassy Carbon Moulding Tools—Analysis of Subsurface Damage

In order to obtain information on possible influences of fine grinding on the final machining of Glassy Carbon forming tools, a forming tool was ground using an Aka Piatto 1200+ (Akasel A/S, Roskilde, Denmark) diamond grinding pad and subsequent examination of the subsurface region by TEM microscopy. During grinding, 20 µm of the original surface (delivery status) were removed. The roughness *Ra* dropped from 240 nm to roughly 40 nm. Since damage to the edge zone of the Glassy Carbon tool surface were suspected, the aim of this investigation was to obtain information about the atomic structure of the Glassy Carbon forming tools at the surface edge and subsurface zones. The investigation took place prior to the moulding experiments. Since the grinding process brings the most energy into the material and thus has the greatest potential for damage, it was not necessary to consider this analysis after polishing again. Besides alterations in the subsurface region, an analysis of the elemental composition of the Glassy Carbon material was also possible by means of TEM. The evaluation of the elements provides information about possible impurities before the press tests.

**Figure 6:**
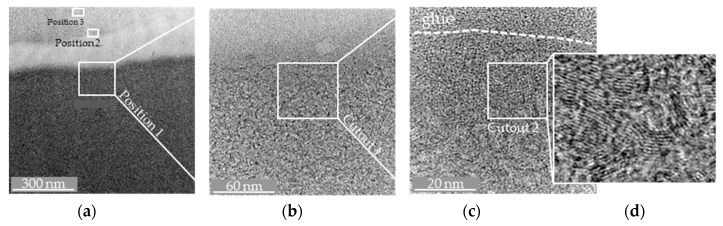
(**a**) Overview on the TEM-measurement area on the wedge-glued Glassy Carbon specimen; (**b**) Magnification at *Position 1*; (**c**,**d**) Further Magnification of the subsurface region and cut-out of the microstructure.

After the preparation of sandwich-glued sample surfaces by wedge grinding (bonding of the interesting surface against itself, that is, Glassy Carbon glued to Glassy Carbon in order to minimise interfering artefacts), the presumed damage zone was mapped by TEM. Figure 6 shows an overview of the more closely examined points of the Glassy Carbon sample. Three positions were examined in more detail. *Position 1* and *Position 2* are each very close above and below the preparation-induced adhesive gap, *Position 3* has been taken somewhat away from the adhesive gap in the very thin, yet near-surface volume of the sample.

Already in this illustration (Figure 6), it can be seen that possible damage to the edge zone of the Glassy Carbon surface caused by the pre-grinding manufacturing process has no major effect on the structure of the material. In order to make the atomic structure of the Glassy Carbon even more visible, a section of *Position 1* (Figure 6b and *Cut-out 1*) has been further enlarged (Figure 6c). The dashed line shows the boundary layer between the adhesive surface and the Glassy Carbon surface. Since the atomic radius of a carbon atom is about 0.1 nm, a further section has been enlarged (Figure 6d, *Cut-out 2*). The atomic structure of the Glassy Carbon is thus clearly visible and no damage to the edge zones could be detected during pre-grinding. The other sections examined (*Position 2* and *Position 3*) confirmed this result.

**Figure 7:**
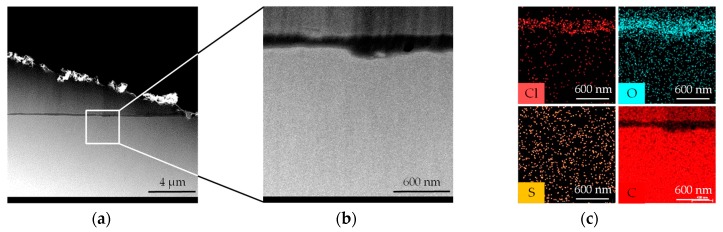
(**a**) Scanning TEM (STEM) measurement for analysis of density alterations in the subsurface region; (**b**) Magnification of STEM measurement spot; (**c**) Energy dispersive X-ray spectroscopy (EDX) analysis of material composition in the magnified area.

Further investigation methods included the STEM- (Scanning TEM: contrast shaping is based on inelastic scattering, that is, density differences are represented in contrast) and EDX-mode (element analysis). The STEM studies confirm the findings of the previous subsurface analysis. The result does not show any density deviations from the sample edge zone to the interior (Figure 7a,b). A homogeneous contrast formation is provided over the whole extent of the examination section.

In the EDX spectrum of the sample area (Figure 7c), only O (oxygen), Cl (chlorine) and S (sulphur) were present in addition to C (carbon). O and Cl were found in the area of the preparation adhesive. These elements are major components of the adhesive used for bonding (Uhu^®^ Plus Endfest 300; 2-component-epoxy-adhesive). S was weak on the entire sample (due to its homogeneous distribution it had probably reached the TEM sample surface from the atmosphere). Within the scope of EDX detection sensitivity (element dependent, typ. approx. 0.1–1%), the sample surface does not differ from the sample volume. None of the found elements find their origin in the Fused Silica since no moulding experiments were performed until this point

Summarising, the process step that induces the highest mechanical load during surface finishing, that is, grinding, does not affect subsurface damage or other structural changes in the microstructure of the Glassy Carbon samples.

### 3.2. Preliminary Polishing of Glassy Carbon Moulding Tools—Analysis of Material Removal

In order to manufacture optical surfaces, polishing provides the following features: low surface roughness, retention of shape accuracy and reduction of subsurface damage [25]. Since the grinding step did not lead to a damaged subsurface region and shape accuracy is not in focus of this study, there is no minimum height reduction mandatory. Nevertheless, it is very important to observe material removal performance on Glassy Carbon of different abrasives. Polishing is known to be a very sensitive process with a comparatively low process understanding. Complex chemical interactions prevent analytical predictions of the machining result.

Under constant process variables (see Figure 5, rotation speed *Ω* = *ω* = 150 min^−1^, contrary rotation; contact pressure *p* = 75 kPa), different polishing abrasives were tested:

- 6 µm diamond grain suspension (6 µm D.)
- 1 µm diamond grain suspension (1 µm D.)
- 0.25 µm diamond grain suspension (0.25 µm D.)
- 1 µm cubic boron nitride grain suspension (1 µm cBN.)
- 0.05 µm colloidal silicon dioxide OPS (oxide polishing suspension) (0.05 µm OPS)

**Figure 8:**
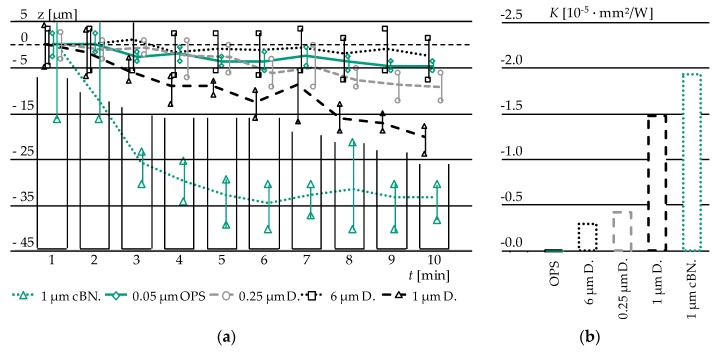
(**a**) Comparison of different polishing abrasives and their material removal expressed as height reduction over time; (**b**) Evaluation of *K*-Factor for different abrasives.

The results of the height reduction *dz* over a time increment of *dt* = 1 (polishing) min are displayed in Figure 8a. Based on the Preston hypothesis (1) and considerations of the relative velocity (2) and (3) the empirical constant K can be derived (Figure 8b).

Clearly discernible differences in the material removal behaviour can be seen. Despite 6 µm D., the removal rate is directly proportional to the grain size of the utilised abrasive. A possible explanation for the low removal of 6 µm could be the “rolling” of the grain over the Glassy Carbon surface, combined with micro ploughing instead of micro chipping. The incremental removal of 0.05 µm OPS was expected, since it is widely used in micro electronic industry for the last finishing step.

### 3.3. Polishing of Glassy Carbon Moulding Tools—Analysis of Achievable Surface Roughness

**Figure 9:**
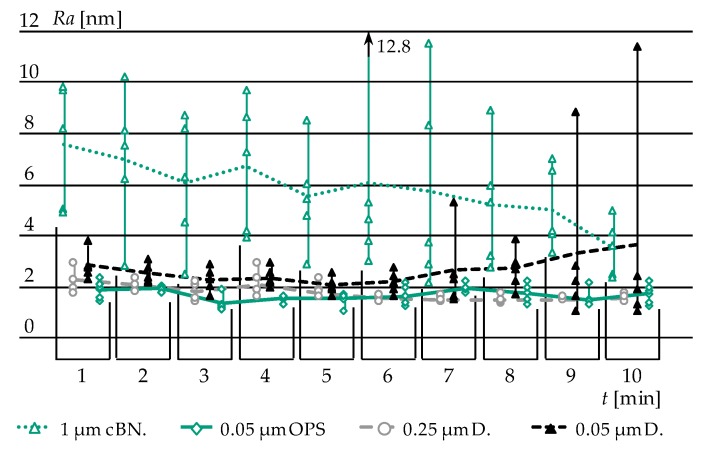
Comparison of different polishing abrasives and their performance in terms of decreasing surface roughness.

For the manufacturing of optical surfaces, an adequately low surface roughness is crucial. Arithmetic mean values *Ra* of below than 5 nm are mandatory. Based on the findings in Section 3.2, the different polishing abrasives are evaluated regarding their ability to achieve high surface quality and integrity. The process parameters were kept constant, the roughness measurement was carried out by White Light Interferometry (WLI) on five points of the Glassy Carbon surface. Prior to polishing (*t* = 0), fine grinding down to a *Ra* value of 8 to 9 nm was applied. The results are displayed in Figure 9 and in the following numeration:

- 1 µm cubic boron nitride grain suspension (1 µm cBN.):
  In the beginning, the polishing led to slight improvement of the surface roughness with a comparatively high spread of the measurement data. The spread increased further after five minutes of machining. At the same time, “orange peel” as a typical polishing damage was visible [26]. In order to avoid this effect, the contact pressure *p* was reduced form 75 kPa to 60 kPa. In the following, the orange peel effect was reduced and low *Ra* values were achieved. Nevertheless, this abrasive does not lead to a surface that would be accepted in the optical industry.
- 0.25 µm diamond grain suspension (0.25 µm D.):
  This polishing agent performed well from the beginning. A further improvement after two minutes of polishing was not observable. The produced surfaces show high integrity (homogeneous polishing patterns) and arithmetic mean roughness values of 1–2 nm. 0.25 µm D. is capable of producing optical surfaces.
- 0.05 µm diamond grain suspension (0.05 µm D.):
  A decrease in diamond grain size was suspected to lead to even lower roughnesses. The outcome could not prove this assumption. Instead, a significant “over polishing” effect was observed, that is, beginning from minute 7, the spread of the measurement data rose. Even by neglecting this circumstance, 0.05 µm D. did not achieve better results than 0.25 µm D.
- 0.05 µm colloidal silicon dioxide OPS (oxide polishing suspension) (0.05 µm OPS):
  OPS performed very well, leading to both low roughness and low spread. Thus, this polishing agent is able to fulfil the demands of optics industry.

With these results, it possible to layout process chains for the manufacturing of Glassy Carbon moulding tools for the replication of Fused Silica.

### 3.4. Topology Generation on Glassy Carbon Moulding Tools—Analysis of Topology

In order to study the influence of the Glassy Carbon surface finishing on its wear behaviour during Fused Silica moulding, it needs to be produced. For these investigations, three different surface finishings that led to different surface topologies were the centre of interest:

- Best possible automatically producible Glassy Carbon surface (Case study A).
  For this case, a surface finish with 0.25 µm D. was chosen (see results in Section 3.3 and Figure 9).
- Glassy Carbon surface with polishing damages (Case study B).
  The orange peel of the 1 µm cBN machining will not lead to damage error-free optics but it is assumed that this topology will lead to alterations in wear behaviour.
- Glassy Carbon surface from specialist in mould manufacturing (Reference, Case study 0).
  Aixtooling GmbH (Aachen, Germany), a specialist for the industrial fabrication of moulds for PGM, took over the finishing step of the Glassy Carbon tools as a reference sample. The exact machining procedure is confidential.

The process chains of mould manufacturing for the case studies are displayed in Table 2. Process step 1 is a grinding procedure (water as cooling liquid), while step 2 and 3 are carried out by polishing and their dedicated polishing agents.

After completing the process chains, the average of five arithmetic mean roughness *Ra* values of Case A reached 1.7 nm. The roughness value of the samples for Case B was considerably higher (*Ra* = 4.5 nm). In contrast to the other two process chains, the Case 0 shows no process of final polishing (Table 2). The samples for Case 0 were pre-polished for two minutes with 1 μm diamond suspension and subsequently submitted to the company Aixtooling for final manual polishing. Prior to this, the average *Ra* after pre-polishing 3 nm. After the final polishing of the samples by Aixtooling, the roughness dropped to a value of *Ra* = 2 nm.

Since special emphasis was placed on the desired diversity of the characteristic global topology during the manufacture of the Glassy Carbon mould pairs, this was checked by means of large-area WLI stitchings, performed on a Bruker Contour GT (Bruker Corp., Billerica, MA, USA). The field examined by the stitching method was circular and had a diameter of 10 mm corresponding to the contact surface of the Fused Silica glass preforms. This macroscopic view on the three forming tool pairs showed that the surface topology of Case B, which was finally polished with a 1 μm cBN suspension, differed strongly from the other two sample pairs. The forming tool pairs produced with the 1 μm cBN suspension showed crater-like defects on the surface. Investigations of the craters by white light images showed that the craters had large differences in diameter but the crater depth, with few exceptions, was in the range of about 20–60 nm. Looking at these defects over the entire surface of the sample, an overall picture similar to orange peel is obtained [26]. The crater-like defects can be seen with the naked eye on very close inspection.

**Figure 10:**
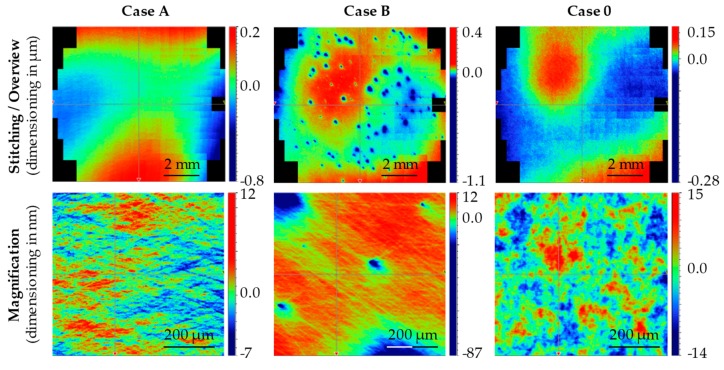
WLI (White Light Interferometry) stitching and in-depth measurements for different surface topologies on Glassy Carbon according to the proposed case studies.

Of the pair of samples, that with the 0.25 μm diamond suspension and that processed by the company Aixtooling GmbH, show a globally homogeneous surface topology that is not disturbed by defects on the stitching images. A comparison of these surfaces is shown in Figure 10 (upper row), which compares the stitchings of the sample pairs of Case A, B and 0. The measurements serve exclusively to illustrate differences in the global characteristic surface topology. Since the sample pairs of Case A and Case 0 do not show any characteristic differences on the stitching images, the different procedure for the preparation of microstructural differences is to be assumed. However, this cannot be represented by the coarse measurement at 2.5-fold WLI magnification. The examination of the two sample pairs at higher magnification (objective with 10-fold WLI magnification) confirmed the assumption (Figure 10, lower row). The Case A samples machine-polished with 0.25 μm diamond suspension show a highly directional microsection structure characterised by superimposition of finest scratches. In contrast, the tools of Case 0 finished manually by Aixtooling have a rather granular isotropic surface.

Hence, all three sample pairs showed differences in their topology and were suitable as input material for investigation regarding the influence of finishing of Glassy Carbon forming tools on wear behaviour during Fused Silica moulding.

### 3.5. Moulding of Fused Silica—Analysis of Wear

This section deals with the main findings of this publication. The Fused Silica moulding was carried out on a Toshiba GMP 207HV, while the geometrical circumstances of moulds (Glassy Carbon, Φ 34 mm) and glass preform (Fused silica, Φ 10 mm, 5 mm height) as well as the process parameters were equal to those for the investigations of Dukwen et al. [27] (i.e., moulding temperature 1360 °C, 2 kN moulding force, 2 min hold time). The evolution of wear phenomena and their dependencies on the surface finishing were observed with several measurement technologies:

- Light Microscopy (Leica S9D, Leica Microsystems GmbH, Wetzlar, Germany);
- Atomic Force Microscopy (DME Type 2329 integrated in Sios NMM; Semilab Germany GmbH, Braunschweig, Germany and SIOS Messtechnik GmbH, Ilmenau, Germany);
- Scanning Electron Microscopy (Zeiss NEON 40 EsB, Carl Zeiss AG, Oberkochen, Germany).

The following structure is leaned on this order.

#### 3.5.1. Light Microscopy

**Figure 11:**
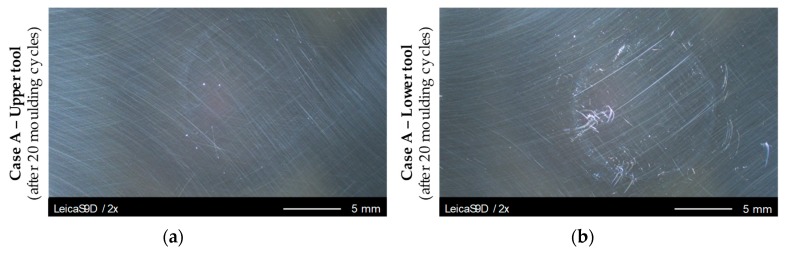
Light microscopic inspection of Case A moulding samples after 20 moulding cycles: (**a**) Case A—Upper tool; (**b**) Case A—Lower tool.

An overview evaluation of the Light Microscopic images of the Glassy Carbon surface had shown that the forming tool pairs of all cases show different degrees of wear. This knowledge does not only refer to the differently machined tools, also differences within the cases, that is, certainly machined tools are recognisable. The comparison of a mould tool set (Case A) shows differences between the upper and lower mould. In comparison with the upper mould, the lower mould shows considerably stronger wear phenomena, for example, load traces. Figure 11 shows a comparison of the microscopic images of the moulds after 20-fold moulding of Fused Silica.

The load traces occur mainly within the glass contact area in the form of scratches, grooves or streaks. These formations can spread up to a few millimetres; they can also be seen without a microscope. In most cases these defects of the surface are not an isolated phenomenon but rather an accumulation of defects in an agglomeration can be observed.

Figure 11b clearly shows such a formation. One of the described formations is located on the left edge of the glass contact area, which can be easily recognised by a slight circle or wreath drawn on the sample that also shows minor discoloration effects. If a single scratch is considered, it is noticeable that the shape of the defect often has an oscillating shape (vibration lines), possibly according to the servo that provides the moulding force. Straight-line formations are less common.

All cases show more or less the same behaviour, especially in terms of differences between upper and lower tool. It is remarkable that the lower tool of Case B (cBN treatment) exhibits a much wider and easily visible wreath at the edge of the glass contact area.

#### 3.5.2. Atomic Force Microscopy

**Figure 12:**
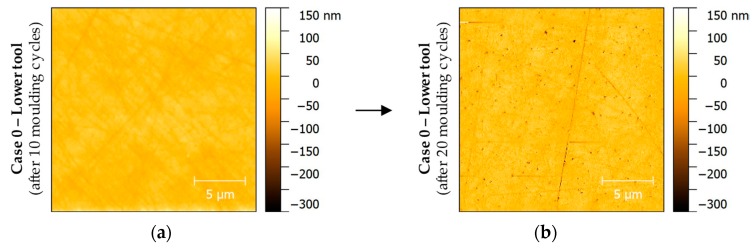
Atomic force microscope (AFM) measurements of Glassy Carbon mould tool surfaces of Case 0: (**a**) Lower tool after 10 moulding cycles; (**b**) Lower moulding tool after 20 moulding cycles.

On a more detailed view, the wear phenomena mentioned above can be consolidated by means of AFM measurements. The measurements were carried out for the initial state of the tool surface and after 10, 20 and 30 moulding cycles, respectively. In that way, the evolution of wear was observable. One of the first findings was the relatively rapid increase in defects after ca. 10 moulding cycles. While up to that point, most of the surface remained unaltered, the AFM plots of 20-fold moulded surfaces showed breakout, build-ups (adhesion) and further scratches for all cases examined. For illustrating these results, AFM images of Case 0 were chosen to be displayed Figure 12.

Both figures show a similar measuring range close to the centre point of the moulding tool, that is, they were placed in the glass contact area. First of all, it should be mentioned that Figure 12a (mould after 10 moulding cycles) does not show any significant differences to the unpressed image. However, Figure 12b shows a massive accumulation of small breakouts, only a few 100 nm in size, from the Glassy Carbon surface. The depth of some of the grooves caused by the manufacturing process has also increased.

**Figure 13:**
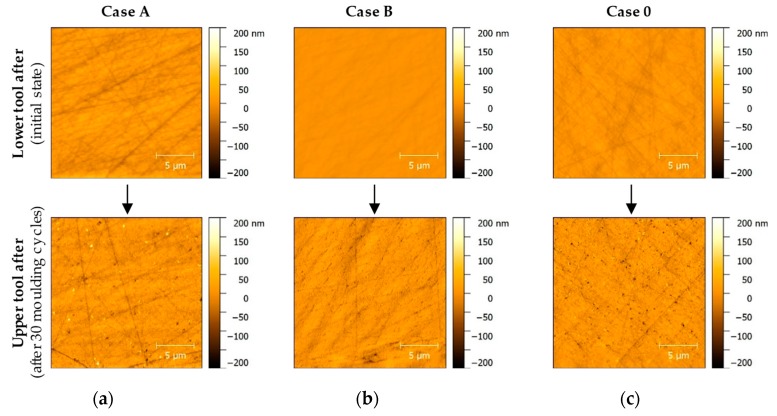
Comparison of the different surface finishing cases: (**a**) Case A; (**b**) Case B; (**c**) Case 0.

The cases show different wear behaviour but the fact that the lower tool degrades more significantly than the upper tool validates the findings of the Light Microscopy. Figure 13 shows an overview of the wear phenomena evolution close to the tool’s centre point of the three forming tools in comparison to the initial surface state.

**Figure 14:**
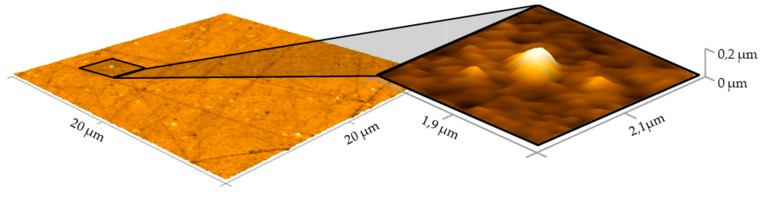
Magnification of an adhesion defect in real aspect ratio.

The lower Case 0 Glassy Carbon mould, which already showed signs of surface wear after 20 moulding cycles (Figure 12), now shows a further form of defect (Figure 13c, bottom row). In addition to the already existing breakouts, there were also sporadic adhesions. The adhesions have a height of up to 200 nm. Defects on the surface of the Case B sample are now also recognisable (Figure 13b, bottom row). These indicate numerous, homogeneously distributed, elongated breakouts from the Glassy Carbon surface. There are no adhesions as in the lower tool sample of Case 0. The imperfections are up to several micrometres long and have a depth of 100–200 nm. The last measuring field, which is located on lower sample Case A, shows the strongest signs of wear (Figure 13a, bottom row). Here, the largest (up to 0.6 μm) adhesions are recognisable. Outbreaks of this magnitude are also represented. The adhesions reach heights of up to 200 nm, also imperfections show depths of the same size, respectively. Figure 14 shows a measurement field of the lower Case A mould in a three-dimensional view. The aspect ratio of the axes in this view is adapted to reality.

The detail section shows an adhered particle. In this illustration, the connection of the particle with the Glassy Carbon surface is clearly visible. This study suggests an adhesive bond of Fused Silica, although the atomic composition cannot be assessed by means of AFM. A dimensional measurement by the software “*Gwyddion*” showed a maximum height of 200 nm and a maximum diagonal of 600 nm (elliptical but nearly circular geometry of the adhesion). The position of several adhesions cannot directly be assigned to previous breakouts or scratches from the finishing procedure.

As the photos of the Light Microscopy examinations already suggested, the AFM photos showed differences in the wear phenomena of the upper and lower mould tool after moulding of Fused Silica. While the upper moulds still showed minor signs of wear, the state of wear on the lower moulds was significantly higher. This effect was visible for all surface topologies, that is, for all case studies.

**Figure 15:**
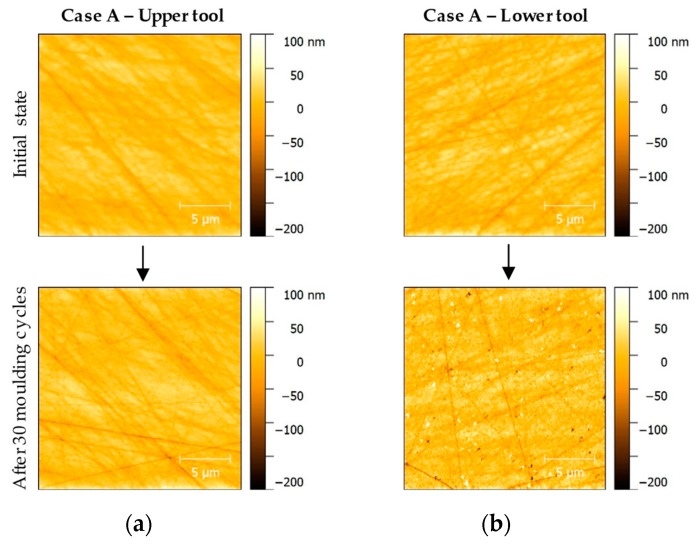
AFM inspection of moulding samples in initial state and after 30 moulding cycles: (**a**) Case A—Upper tool; (**b**) Case A—Lower tool.

The comparison of the AFM measurements of the upper forming tool of Case A and the surface development of the dedicated lower tool illustrates the different wear behaviour (Figure 15). The upper mould remains largely free of signs of wear, no breakouts or alteration of the surface topologies induced by surface finishing can be observed. The lower mould exhibits significant wear as mentioned before (Figure 13 bottom row).

#### 3.5.3. Scanning Electron Microscope

In addition, the Glassy Carbon forming tools—with the exception of the Case 0 sample pair—were measured with SEM (Scanning electron microscope) microscopically on their surface after each 20 pressing cycles. Since the SEM investigations conducted by Dukwen et al. [19] showed up to 0.5 μm large damages of the Glassy Carbon surface after 20 pressings, the same images should be carried out for reasons of direct comparability. In addition, EDX analyses were performed to determine the material composition of defects or anomalies, which were not examinable by means of Light Microscopy or AFM.

The SEM images confirmed the AFM measurement results. After 20 moulding cycles, none of the Glassy Carbon forming tools showed the suspected, numerous punctiform adhesive adhesions [19]. This could provide different advice on the influence of the surface finish on the wear behaviour, since the samples of Dukwen et al. reached a minimum roughness of *Ra* ~ 5 nm, while the tools used for this publication reached partial *Ra* values of below 2 nm.

**Figure 16:**
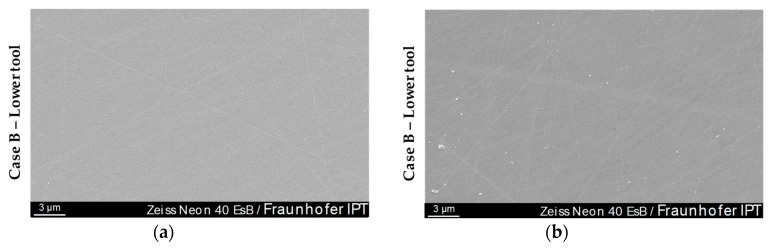
Scanning electron microscope (SEM) inspection of Case B Glassy Carbon moulding tools (after 20 moulding cycles): (**a**) and (**b**) name different measurement spots in the glass contact area.

Figure 16 shows a SEM image of the glass contact area of the lower Case B moulding tool after 20-fold moulding of Fused Silica glass. The grooves on the surface caused by the manufacturing process are also clearly visible. Furthermore, slight contamination of the sample surface is visible but this does not testify to the suspected adhesive adhesions (Figure 16, bottom left corner).

**Figure 17:**
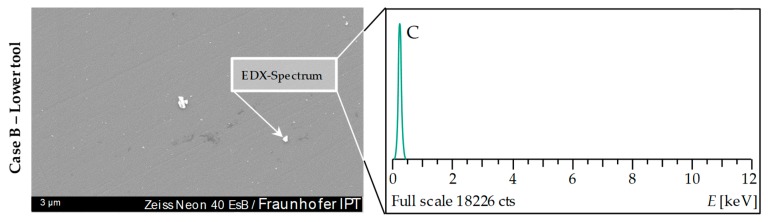
Carbon imperfections on Glassy Carbon surface of Case B tool samples and corresponding EDX measurement.

An EDX element analysis was performed in order to find the origin of the contaminations. The measurement revealed that the light-coloured contaminants present on the Glassy Carbon consist of pure carbon (C). The spectrum of elements showed virtually no traces of glass elements (e.g., silicon (Si)). Figure 17 shows the evaluation of such a particle with the associated EDX plot.

A possible explanation for the imperfections could be graphite contaminations of the graphite mould dies, which embrace the Glassy Carbon moulds. Thermal expansion effects during heating and cooling of the mould system could have led to material subversion in the gap between graphite and Glassy Carbon components. Other particles investigated showed slight material impurities. One of these was the mineral wollastonite (CaSiO_3_). Even very small amounts of silicon carbide were found. However, these shares were insignificantly small. Furthermore, the size of the particles varied greatly. There were particles that were only fractions of a micrometre in size but also particles up to 30 μm and occasionally larger, even outside of the glass contact area.

**Figure 18:**
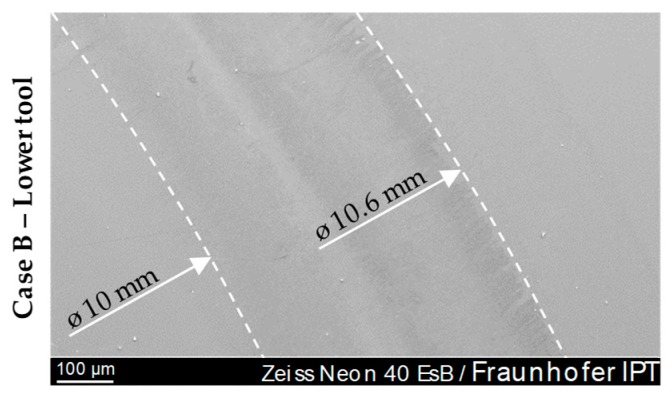
Alterations in the glass transition zone of Case B moulding tools.

Considerations of the transition area, that is, the area that has emerging glass contact due to the compression of the cylindric glass preform, showed no special features. The image of the surface was similar to that of the glass contact area. Figure 18 shows the transition area. The ring- or wreath-shaped circle is clearly visible.

**Figure 19:**
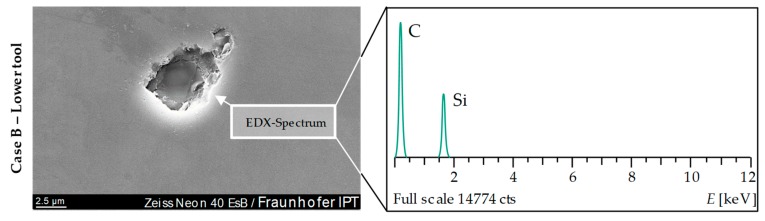
Fused Silica agglomeration on Glassy Carbon tool surface of Case B.

As already mentioned above, anomalies (specific individual defects) were documented. These defects were found on samples prepared by cBN polishing (Case B). In most cases, these were break-outs from the Glassy Carbon surface in the annular transition zone (Figure 18). Parts of silicon (Si) were found in these defects as well. These Fused Silica agglomerations settled in or close to outbreaks. Figure 19 shows such an isolated defect.

**Figure 20:**
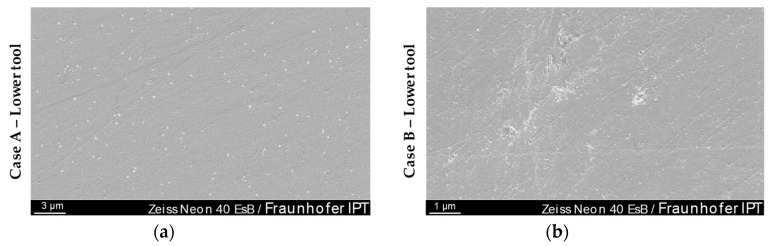
Alterations of the Glassy Carbon tool surface of 30 moulding cycles analysed by SEM: (**a**) Case A—lower tool; (**b**) Case B—lower tool.

Further SEM investigations after 30-fold Fused Silica moulding cycles confirmed the wear patterns of the AFM image that occurred after this moulding interval (Figure 13). Figure 20 shows a SEM images of Case A and B on a centre-near position.

The signs of wear visible on the AFM measurements can clearly be retrieved on the SEM images. The distribution, size and appearance are similar to those of Figure 13. On closer inspection of the image, it can be seen that the adhesions are preferentially but not generally located in outbreaks or peaks of the Glassy Carbon surface. An EDX element analysis showed that the punctiform wear phenomena, shown brightly in Figure 20a, are composed of Fused Silica. As stated before, no glass adhesion could have been found on the cBN treated moulds (Case B).

## 4. Discussion

The publications of Dukwen et al. [19,27] form the foundation of the approach to put Fused Silica moulding into an industrial context. This research dealt with preliminary investigations of the wear behaviour in Fused Silica forming by Precision Glass Moulding. In particular, the wear behaviour of the Glassy Carbon forming tools in relation to the process parameters used in the forming process and tribological conditions were considered. Differences in wear behaviour between the upper and lower moulds had already become known at this point. The investigation pointed to higher wear of the lower moulds.

**Figure 21:**
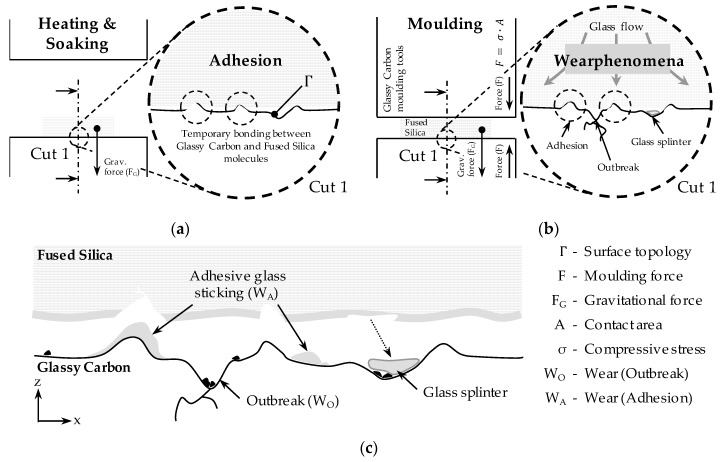
Extended wear evolution model of Fused Silica moulding with Glassy Carbon moulding tools (based on [27]): (**a**) Temporary bonding between the contact partners; (**b**) Conditions during moulding phase; (**c**) Wear result after moulding and detachment of the moulded glass component.

The investigations carried out in this publication make use of these previous findings and extend them based on more in-depth material qualification and extent measurement effort in order to gain more data on wear evolution. Dukwen et al. explain these increased wear phenomena occurring on the lower forming tools due to so called static adhesion [27]. Since the Fused Silica glass samples are already in contact with the Glassy Carbon mould during the heating and soaking phase, leading to temporary bonds between the two materials. There is no contact between the upper mould and the Fused Silica preform during the heating phase. Hence, no static adhesion is formed on the upper mould. In the moulding phase, these static bonds between the glass and Glassy Carbon molecules partly remain and lead to cohesion fractures within the glass volume. Figure 21 illustrates this process schematically and extends the model of static and dynamic adhesion.

The cohesion fracture that leads to glass adhesion is said to take place due to the change in shape of the glass in the horizontal direction and the resulting shear loads, combined with higher stresses in the area of imperfections of the opposed Glassy Carbon surface, implying a significant influence of its surface finishing on wear behaviour. The stronger bonds between the Fused Silica and the Glassy Carbon cause the glass to break out of the sample and adhere to the Glassy Carbon as adhesive particles (Figure 21b).

This study revealed that the glass adhesions are not only found on asperities or breakouts on the glassy Carbon surface (Figure 21c), that is, a high mechanical load is no singular reason for adhesive wear. Furthermore, the fracture resistance of Fused Silica is much higher than for other glass types (especially for low strain rates as performed in this study) [28]. This implies there might be several reasons for Fused Silica adhesions. Since the differences of upper and lower tool wear strongly depend on the glass contact time, also chemical interactions must be taken into consideration.

Besides that, the observations showed that existing imperfections (e.g., outbreaks, outbroken particles, glass adhesions and glass splinters without significant bonding to a surface) reinforce the degradation during the following moulding cycles (Figure 21c). Since all of these imperfections are supposed to have edges, they are like abrasive particles that scratch the surface of the Fused Silica sample when the glass expands and vice versa. From this point of view, the status of the surface finish can be seen as the initial state of a chaotic system, since the formation of breakouts and particles affect all following states.

In order to put the findings into functional relationships, the main influence factors on the wear phenomena (*W*—wear, *W_O_*—wear in the form of outbreaks, *W_A_*—wear in the form of adhesions) can be expressed as follows:

(4) $$W=W_{O}+W_{A}$$

(5) W=W(σ,T,t,Γ)

Generally, the wear can be expressed as the sum of outbreaks and adhesion effects (Equation (4)). The main influence factors of both phenomena are contact pressure *δ*, moulding temperature *T*, hold time *t* (including heating and soaking sequence) and surface topology *Γ*. According to theoretical considerations, specific adjustments of the process parameters would lead to a decrease in wear (Equation (6)).

(6) $$\underset{\sigma \to 0}{\lim}W=\underset{T\;\to 0}{\lim}W=\underset{t\;\to 0}{\lim}W=0$$

Unfortunately, these trivial relations cannot be realised in terms of a real production process, due to the following reasons [28,29]:

- Decreasing moulding force F or contact pressure *σ* would lead to less mechanical load but the desired glass flow comes to a halt.
- Decreasing moulding temperature would lead to less activation of wear processes but also to a higher viscosity of the glass, which would lead to higher mechanical load and an induction of stresses and fractures (The moulding temperature of 1360 °C equals a viscosity of *η* = 10^10^ dPas (Figure 3Right), which marks a process border already).
- Decreasing contact time (especially during heating and soaking phase) would overcome the issues of temporary bonding but is not realisable in the existing machine set-up.

In summary, the parameters mentioned above cannot be seen as independent variables, since they are interconnected by means of the viscoelastic behaviour of glass. By a variation of the process parameters, only incremental progress in terms of wear reduction can be expected.

Nevertheless, this study put a strong focus on the influence of the surface finishing of the Glassy Carbon tools, leading to different surface topologies (*Γ*). It was assumed that the Glassy Carbon moulding tools of Case A, which had been finished by the 0.25 μm diamond suspension and had the best quantitative surface characteristics in terms of *Ra* value, would show the least wear phenomena after the moulding tests. This assumption was based on the general circumstance that the relatively best surface topology would have the least pronounced micro-contact sites. This should result in less shear stress on both contact partners. Likewise, the very smooth surface texture should result in fewer breakouts from the Glassy Carbon surface due to the manufacturing process. This was also expected to reduce wear behaviour. In contrast to the very homogeneous surface of the samples of Case A, which was characterised by superimposition of very fine craters, a slightly worse wear behaviour was expected from the sample surface prepared by the company Aixtooling, a specialist for tool manufacturing for PGM (Case 0). Since these samples on the WLI images showed a rather coarse-grained, isotropic microstructure, which also showed good roughness values, a slightly higher wear was assumed. Furthermore, it was assumed that the coarser microstructure would provide more surface area for the viscoelastic Fused Silica flow and thus lead to greater shear stress in the contact zone. The samples of Case B, which had the worst roughness values and was unsuitable for the production of optics due to craters on the surface, showed, contrary to expectations, the least signs of wear after the forming tests. Only fine, elongated grooves were visible. It was suspected that the insertion of the craters into the surface would lead to varying contact stresses on the material, which could have had an effect on the wear behaviour.

It is possible that the wear behaviour, which was reflected by the many small elongated grooves, was related to possible variations of the contact stresses. However, this connection is only speculative. As already mentioned above, the best wear behaviour was expected from the Glassy Carbon samples processed with 0.25 μm diamond suspension. However, the observation was different. The AFM and SEM images showed up to 1 μm large build-ups and adhesions on the Glassy Carbon surface. The largest breakouts of all three measured surfaces were also documented on the lower tool of Case A. The pair of samples, which had been finished by Aixtooling (Case 0), unexpectedly showed signs of wear already after 20 moulding cycles of Fused Silica, in contrast to the other pairs of moulds. However, these phenomena were limited to small breakouts from the surface that were only a few 100 nm in size. After 30-fold forming of Fused Silica glass, the breakouts increased and were similar to those of sample C6. In addition, there were slight adhesions, which were also only a few 100 nm in size.

**Figure 22:**
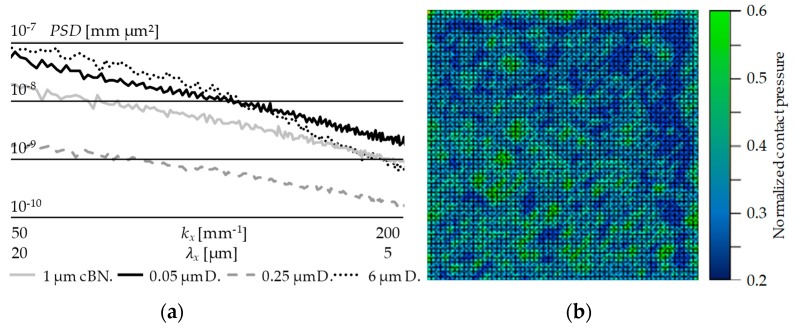
(**a**) PSD (Power spectral density) progressions for different surface finishings; (**b**) First attempts of FEM simulation using realistic surface profiles of Glassy Carbon surfaces in Fused Silica contact.

In summary, the hypothetical existence of an influence of the surface finishing of Glassy Carbon moulding tools on the wear behaviour during Fused Silica moulding was proven correct. Nevertheless, the clear correlation of mechanisms of wear evolution remain unsolved, since the expectations of the different surface topologies regarding their wear behaviour were proven incorrect. Therefore, the target of this publication—the derivation of measures to optimise the tools’ service lifetime—was not achieved due to a lack of deductive explanation approaches. Before negating the general assumption of adhesion evolution induced by shear stresses and cohesion fracture, a possible drawback of the experimental conduction should be taken into account: This could be the surface assessment by means of simple profilometric values. Since profilometric values cannot distinguish between imperfections on different dimensional scales (i.e., a surface with several small infinite scratches can lead to the same Ra value as a flawless surface with a singular crater), it could turn out that a clear differentiation between “good” and “bad” surfaces (e.g., Case A and Case B respectively) needs to be rethought. A possible solution in terms of a more sophisticated surface qualification could be an analysis by means of “Power spectral density” (PSD), an algorithm based on a Fourier transformation of the surface profile [30] (Figure 22a). A clear distinction between scratches, pits, outbreaks, adhesion, agglomerations and so forth, could be made possible. A PSD reprocessing of the collected raw data will be conducted in the near future in order to find more reliable correlations between surface finish and degradation of the tools.

Furthermore, an FEM simulation could provide further hints on the contact situation [31]. In terms of convergence on the real problem, extracts of the real surface topologies should make up the tool interface in the simulation model (Figure 22b).

## Figures and Tables

**Table 1:** Characteristics of Fused silica, type Suprasil 300 [5,14].

Properties	Fused Silica (Suprasil 300)
Density *ρ*	2.7 g/cm^3^
Trans. temperature *Tg*	~1080 °C
Thermal expansion α	0.5 × 10^−6^ 1/K
OH^−^ impurities	<1 ppm
Maximum work temperature	950 °C
Moulding temperature	1360–1400 °C

**Table 2:** Machining protocol for surface topology generation.

Sample:	Case A			Case B			Case 0		
Step	1	2	3	1	2	3	1	2	3
Handling	Aka ^1^	1 µm D.	0.25 µm D.	Aka	1 µm D.	1 µm cBN	1 µm D.	1 µm D.	Aix ^2^
*t* (min)	3	2	2	3	2	6	3	2	Aix
Rotation *Ω* (min^−1^)	150	150	150	150	150	150	150	150	Aix
Rotation *ω* (min^−1^)	150	150	150	150	150	300	150	150	Aix
Contact pressure *p* (kPa)	75	75	75	75	75	60	75	75	Aix

^1^ Aka Piatto 1200+ diamond grinding pad, ^2^ Aixtooling GmbH.
